# Photo cleavable thioacetal block copolymers for controlled release†

**DOI:** 10.1039/d1py00514f

**Published:** 2021-06-08

**Authors:** Yongjun Men, Tobias G. Brevé, Huanhuan Liu, Antonia G. Denkova, Rienk Eelkema

**Affiliations:** Department of Chemical Engineering, Delft University of Technology van der Maasweg 9 2629 HZ Delft The Netherlands r.eelkema@tudelft.nl; Department of Radiation Science and Technology, Delft University of Technology Mekelweg 15 2629 JB Delft The Netherlands A.G.Denkova@tudelft.nl

## Abstract

We present a new light cleavable polymer containing *o*-nitrobenzene thioacetal groups in the main chain. By conjugation to a PEG block, we synthesized block copolymers capable of forming nanoparticles in aqueous solution. We studied drug encapsulation and release using the model drug Nile Red. Irradiation with UV-A light (365 nm) leads to efficient degradation of the polymers and associated burst release of the payload. Unlike other thioacetal and thioketal polymers, these polymers are stable to reactive oxygen species (ROS), preventing non-triggered release. Moreover, the nanocarriers showed low cytotoxicity in cell viability experiments.

## Abstract 2

The *o*-nitrobenzene thioacetal group selectively cleaves upon UV-A irradiation. When incorporated in a block-copolymer, these photoactive groups can be used for controlled release of molecular cargo from polymer nanoparticles.

## Graphical abstract

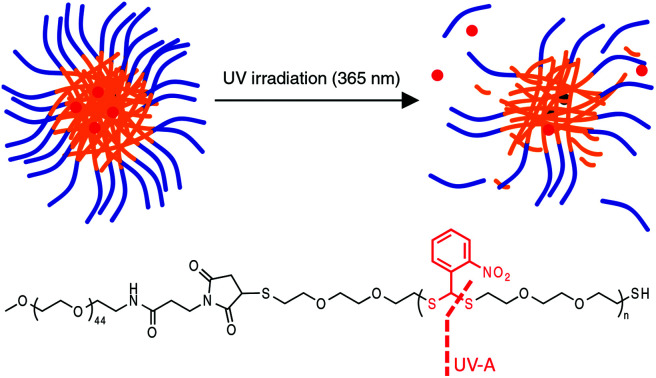

## Introduction

Stimuli-responsive polymers are of great interest in nanomedicine since they allow accurate release of drugs at the disease site, while minimizing side effects to healthy tissues.^1–3^ Such polymers have been used for drug release by encapsulating drugs or directly linking drugs to the polymer side chains.^4–7^ Changes of the polymer physical properties (*e.g.*, the aggregation state) or its chemical structure can lead to drug release, in response to triggers emanating from the body (*e.g.*, pH, temperature, biomarkers, reactive oxygen species (ROS)) or from external sources (*e.g.*, light, heat, magnetic fields, ultrasound).^8–20^ Light has multiple advantages for use as a trigger, including easy and local application, and fast response. These advantages have led to a great interest in photocleavable polymers, which offer the potential for spatiotemporal control over the cleavage of covalently linked prodrugs or the disassembly of block copolymer micelles or vesicles.^21^ Photolabile compounds, or photocages, include *o*-nitrobenzene (*o*-NB), nitro-benzofuran, 6-bromo-7-hydroxycoumarin, quinolone, cyanine, and others.^22–25^ Among these, *o*-NB equipped polymers have been widely investigated due to the versatility of *o*-NB monomers.^26^*o*-NB based linkers and protecting groups undergo photocleavage upon UV-A irradiation (315–400 nm), a process that takes place on a time scale of minutes to several hours depending on the light intensity used and the quantum yield of the light sensitive moiety.^27^

Three approaches have been reported to prepare *o*-NB based polymers.^28^ In the first approach, a *o*-NB-functionalized alkene type monomer is polymerized *via* free radical polymerization. The length of the backbone of these *o*-NB-decorated polymers remains unchanged after cleaving the *o*-NB moieties upon UV irradiation.^29^ An advantage of this approach is the possibility to achieve a high degree of substitution by the *o*-NB moiety. On the other hand, this approach requires complicated monomer synthesis and shows slow drug release rates since the polymer backbone is not degraded, and many *o*-NB moieties must be removed to achieve the desired effect. The second approach is the synthesis of a block copolymer, in which an *o*-NB derivative is used as covalent photocleavable linker between the blocks.^28,30^ The third approach relies on condensation polymerization of *o*-NB derivatives to obtain polymers that include *o*-NB moieties in their backbone.^31^ In this polymer type *o*-NB enables the cleavage of the polymer backbone upon UV irradiation, leading to a fast drug release, since a small fraction of cleaved bonds results in a large decrease in the polymer molecular weight. One example is polymerizing *o*-nitrobenzaldehyde (*o*-NBA) with monomers containing carboxylic acid and isocyanide *via* the Passerini reaction.^32,33^

**Fig. 1:**
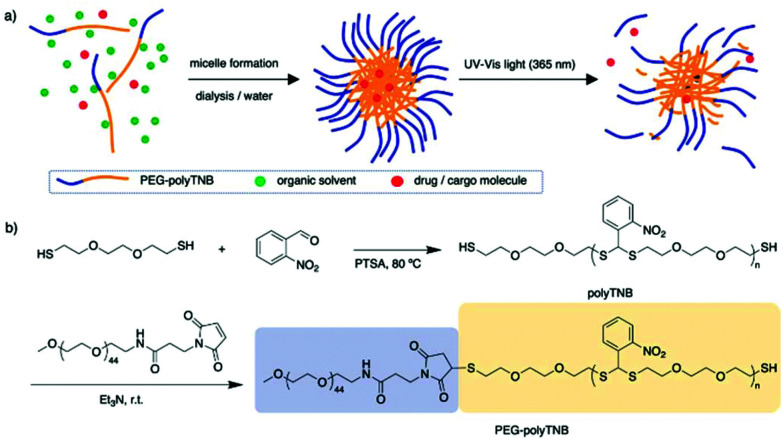
Scheme of PEG-polyTNB nanoparticle assembly and light triggered release. (a) Block copolymer micelle formation and light-triggered drug release; (b) synthesis of PEG-polyTNB block copolymer from dithiol and *o*-nitrobenzaldehyde, followed by thia-Michael addition to PEG-maleimide. PTSA = *p*-toluene sulfonic acid.

In 2010, Murthy *et al.* reported a thioketal-based polymer synthesized by condensation of low molecular weight thiols and ketones.^34^ The thioketal bonds can be easily broken by reaction with ROS while they remain stable in harsh acid and alkaline conditions, providing a possibility for oral drug delivery. In another report, Choi *et al.* prepared a thioacetal *o*-NBA dual-arm photocage that enabled control of the simultaneous release of two payloads linked to a single unit, and the photocage showed a quantum yield of 0.2.^35,36^ Inspired by these reports we anticipated that a thioacetal polymer synthesized through condensation polymerization of *o*-NBA and a dithiol will have photoresponsive properties.^34,37^ Furthermore, any remaining thiol end groups can be easily conjugated to hydrophilic polymers through maleimide click chemistry, to prepare amphiphilic block copolymers that can assemble into nanoparticles and can be used as a photo-cleavable drug carrier (Fig. 1).

## Experiment

### Materials

All the chemicals were bought from Sigma-Aldrich and used without further purification. Benzyl mercaptan (99%), *o*-nitrobenzaldehyde (98%), *p*-toluenesulfonic acid (>98.5%), methoxypolyethylene glycol maleimide (mPEG-maleimide, *M*_w_ = 2 kDa, >90%), Nile Red (>98%).

### Synthesis of polyTNB

A mixture of 3,6-dioxa-1,8-octanedithiol (1.62 mL, 10 mmol), *o*-nitrobenzaldehyde (1.51 g, 10 mmol), and *p*-toluenesulfonic acid (1.7 mg, 10 μmol) as a catalyst was stirred at 80 °C under argon protection for 4 days. The reaction mixture was precipitated into diethyl ether 3 times and the obtained polymer was then dried under vacuum overnight giving polyTNB as a yellow sticky solid (3.29 g, 99%). The ^1^H-NMR spectrum is shown in Fig. S2.†

### Synthesis of PEG-polyTNB

We dissolved polyTNB (250 mg, 52.4 μmol) in chloroform (8 mL), and added mPEG-maleimide of 2 kDa (105 mg, 52.4 μmol) and Et_3_N (1 drop). The reaction mixture was magnetically stirred for 24 h at room temperature and then concentrated *in vacuo* to give an oily product. Deionized water (20 mL) was added to the crude product and stirred for 30 min. The precipitate was removed, and the precipitate was further washed with methanol (150 mL) 3 times. The obtained gray sticky solid was vacuum dried for 48 h. Yield: 320 mg, 90%.

### Preparation of Nile Red loaded nanoparticles

We used a solvent switching method for the preparation of empty (control) polymeric nanoparticles and cargo-loaded nanoparticles. A typical procedure is as follows: PEG-polyTNB (20 mg) was dissolved in a mixture of tetrahydrofuran (THF) and 1,4-dioxane (dioxane) (2 mL, 4 : 1 by volume) with Nile Red (10 μL, 20 mg mL^−1^ in DMSO) in a 15 mL capped vial with a magnetic stirrer. After the compounds were stirred for 30 min at room temperature, we added water at a rate of 1 mL h^−1^ using a syringe pump. The needle from the syringe was inserted into the vial of which the cap was replaced with a rubber septum. 2 mL of water was pumped into the organic solution with vigorous stirring (900 rpm). After the water addition was finished, the suspension was transferred to a dialysis tube and dialysis against Milli-Q water 3 L for 48 hours with changing frequency of every 12 hours.

### Encapsulation efficiency

Encapsulation efficiency (EE%) of dye-loaded nanoparticles was calculated by the following formula:
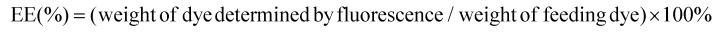


The amount of Nile Red was measured by the fluorescence intensity. We first draw a standard curve by using the fluorescence intensity of a series of variant concentrations (0.001 to 0.1 μg ml^−1^) of Nile Red in tetrahydrofuran (THF) (Ex: 527 nm, Em: 604 nm). To determine the total amount of Nile Red associated with the nanoparticles, the nanoparticle solution was centrifugated to remove the water. Then the aggregated nanoparticles were dissolved in THF, and measured their fluorescence intensity.

### Photo-responsiveness and photo-cleavage reaction

One milliliter of the PEG-polyTNB micelle solution (2.5 mg mL^−1^) was exposed to UV light (365 nm, 90 mW cm^−2^) for 0–15 min in a QS cuvette. At a certain time, 10 μL of the solution was taken out and diluted to 40 times in water for UV and DLS measurements. 20 μL of the solution was mixed with 200 μL THF for GPC measurement. NMR samples were freeze-dried under vacuum (0.01 bar) overnight and re-dissolved in 0.5 mL CDCl_3_.

### ROS-responsiveness

Structural changes of polymers in response to treatment with ROS reagents (H_2_O_2_ or KO_2_) were measured using ^1^H-NMR spectroscopy. To 1 mL of the polymeric micelle solution (2.5 mg mL^−1^) in PBS (pH 7.4) we added H_2_O_2_ (10 M) to form a 500 mM solution or KO_2_ to form a 100 mM solution. After incubation at 37 °C for 24 h, the solution was lyophilized, and the product was studied by ^1^H NMR.

### Dye release behavior

We performed cargo release experiments of Nile Red loaded polymeric nanoparticles in neutral PBS (pH 7.4). The Nile Red release behavior after UV365 light irradiation was investigated by fluorescence measurement. Specifically, 1 mL nanoparticles (2.5 mg mL^−1^) was transferred into a dark QS cuvette. The fluorescence of the release solution at different time intervals was measured at 601 nm emission wavelengths by a microplate reader (excitation wavelength at 530 nm).

### Cytotoxicity of PEG-polyTNB nanoparticles

HeLa cells were kept in DMEM culture medium (Dulbecco's Modified Eagle's Medium, Biowest) supplemented with 10% mL fetal bovine serum (Gibco, Life Technologies) and 1% Penicillin/Streptomycin (100×, Biowest) under humidified normoxic conditions (95% air, 5% CO_2_) at 37 °C. For evaluating the *in vitro* cytotoxicity of the PEG-polyTNB nanoparticles, 2000 HeLa cells (suspended in 200 μL cell culture medium) were seeded in 96-well plates and incubated for three days. 10 μL of PEG-polyTNB nanoparticles or pre-irradiated (8 min) PEG-polyTNB nanoparticles was added to each well, with the final micelle concentration of 0.25 mg mL^−1^. After being incubated for another 24 h, the old culture medium was removed, the cells were washed with PBS twice and 200 μL of fresh culture medium was added to HeLa cells. Another 3 days has been given for the cell to growth before the WST-8 assay (Cell Counting Kit-8, Dojindo Laboratories, Tebu-Bio). For the test, 10 μL of CCK-8 reagent was given to each well and incubated for 3 h, then the absorptance at 450 nm was measured by a microplate scanning spectrophotometer (PowerWave XSTM, Bio-Tek). The surviving fraction (SF) of the HeLa Cells was calculated using the following equation:
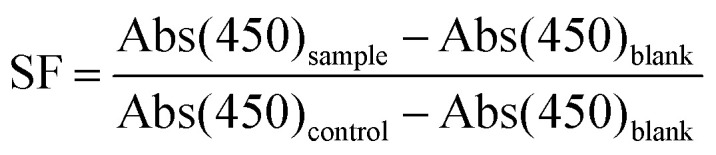
Abs(450)sample is the absorptance at 450 nm for cells with PEG-polyTNB nanoparticles; Abs(450)control is the absorptance at 450 nm for cells with 10 μL of culture medium; Abs(450) blank is the absorptance at 450 nm for vials without addition of CCK-8 reagent.

## Results and discussion

### Synthesis and characterization of polyTNB

**Fig. 2:**
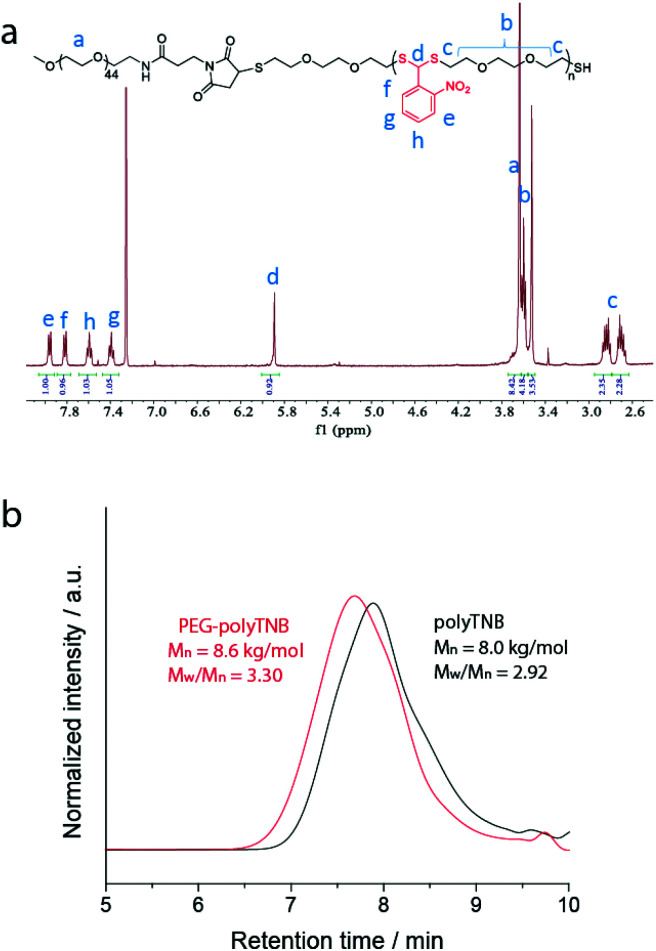
(a) ^1^H-NMR spectrum of PEG-polyTNB in CDCl_3_; (b) molecular weight and dispersity of polyTNB and PEG-polyTNB measured by GPC.

The synthesis scheme of PEG-polyTNB block copolymer is shown in Fig. 1b. 3,6-Dioxa-1,8-octanedithiol and *o*-nitrobenzaldehyde were polymerized to polyTNB in a bulk polymerization using *p*-toluenesulfonic acid (PTSA) as catalyst. The obtained polyTNB shows highly elastic (low glass transition temperature, *T*_g_ = −17 °C, Fig. S1†) behavior due to the ethylene glycol segments in the polymer chain. A peak at 5.89 ppm that belongs to the proton in the thioacetal group could be observed in ^1^H-NMR (Fig. S2†), while the starting material aldehyde peak of *o*-NBA is absent. The molecular weight (*M*_n_) of polyTNB is 8.0 kg mol^−1^ (*Đ* = 2.92, Fig. 2b) determined by gel permeation chromatography (GPC). The broad molecular weight distribution results from the condensation polymerization method. The dried polyTNB was then reacted with an equimolar amount of methoxy-PEG-maleimide assisted by a catalytic amount of triethylamine (TEA) at room temperature for 24 h to form the PEG-polyTNB block copolymer. All peaks in the ^1^H-NMR spectrum (Fig. 2a) correspond to PEG-polyTNB, with integration of the aliphatic peaks suggesting that the major product is the mono-PEGylated polyTNB. The molecular weight of PEG-polyTNB as measured by GPC (*M*_n_ = 8.6 kg mol^−1^, *Đ* = 3.30, Fig. 2b) is somewhat lower than the expected 10.0 kg mol^−1^ (polyTNB 8.0 kg mol^−1^ + methoxy-PEG-maleimide 2.0 kg mol^−1^), likely due to two reasons. First, the radius of gyration of PEG-polyTNB in THF (GPC mobile phase) is different as that of single PEG and polyTNB. Second, a small percentage of polyTNB remained unreacted or conjugated to two PEG chains (PEG-polyTNB-PEG), resulting in a broader dispersity.

### Characterization of PEG-polyTNB nanoparticles

**Fig. 3:**
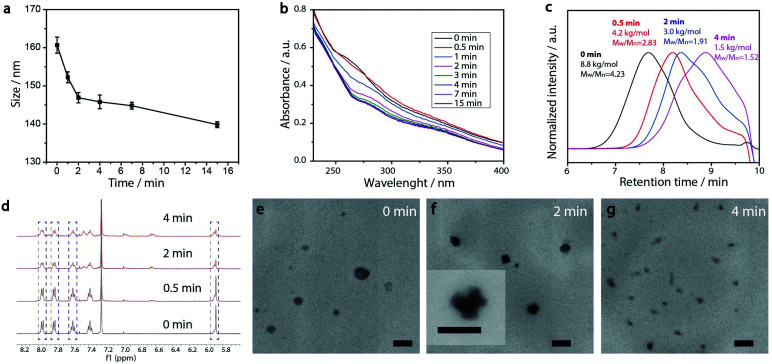
PEG-polyTNB nanoparticle degradation depending on the duration of UV-A (365 nm) exposure. (a) Size changes measured by DLS; error bars represent standard deviation based on three measurements. (b) UV-vis absorption spectra changes; (c) molecular weight and distribution change measured by GPC using polystyrene standards for reference; (d) molecular structure changes measured by ^1^H-NMR (PEG unit was chosen as calibration standard due to no signal change during the reaction); (e–g) morphology changes measured by TEM, scale bar 200 nm.

For the preparation of PEG-polyTNB nanoparticles, we used a solvent switching method. PEG-polyTNB (10 mg) was dissolved in THF/dioxane (vol/vol 1 mL), followed by the slow addition (2 mL h^−1^) of 1 mL H_2_O. The turbid transition (*i.e.* critical aggregation concentration) occurred at 24–25 vol% H_2_O content. The organic solvents were removed by dialyzing the suspension against Milli-Q water for 48 h. The mean size of the obtained nanoparticles was 159 nm (PDI = 0.082) as measured by DLS. The TEM analysis shows that the nanoparticles appear as nearly spherical shapes, with smaller size (∼100 nm) than DLS due to the drying effect (Fig. 3e).

### Light degradation of PEG-polyTNB nanoparticles

PEG-polyTNB nanoparticles are expected to decompose upon UV-A light (365 nm) irradiation resulting from the photolability of the *o*-nitrobenzyl unit. To examine the light-triggered degradation, we exposed 1 mL of PEG-polyTNB nanoparticles solution (2.5 mg mL^−1^) under 365 nm light with an intensity of 90 mW cm^−2^. DLS results show the nanoparticle size decreased from 159 nm to 146 nm in the first 2 min, and slowly reduced to 140 nm in the following 13 min, indicating that the decomposition reaction proceeded quickly in the first 2 min which is consistent with the results of the UV experiment (Fig. 3b). After being irradiated for 15 min, no precipitation observed from the nanoparticle suspension, but shows a slightly decrease of zeta potential that changed from −41.8 mV (before irradiation) to −34.1 mV (UV 0.5 min) and −32.6 mV (UV 4 min) (Fig. S3†). The morphology of nanoparticles changed from a spherical shape to irregular shapes within 2 min of irradiation as was observed from TEM images (Fig. 3e–g). These shape changes might be caused by polymer chain rearrangement induced by backbone breaks. To understand the chemical change of the polymer backbone at the molecular level, we analyzed the nanoparticles by NMR and GPC after freeze-drying of the nanoparticles. ^1^H-NMR (Fig. 3d) shows how the peak at *δ* = 7.59 ppm, belonging to an aromatic proton in the nitrobenzyl group (proton g, Fig. 1a) decreased on average 12%, 34%, and 50% after UVA irradiation for 0.5, 2, and 4 min, respectively (Fig. S4†). Since a small fraction of bond breaking of the polymer will result in a large reduction of the polymer average molecular weight, by only 0.5 min UVA irradiation the average *M*_n_ of PEG-polyTNB drops from 8.8 kg mol^−1^ (*Đ* = 4.23) to 4.2 kg mol^−1^ (*Đ* = 2.83) and further to 3.0 kg mol^−1^ (*Đ* = 1.91, UV 2 min) and 2.5 kg mol^−1^ (*Đ* = 1.52, UV 4 min), as shown in Fig. 3c. We observed that the molecular weight of PEG-polyTNB is different before (8.6 kg mol^−1^, *Đ* = 3.30) and after micellization (8.8 kg mol^−1^, *Đ* = 4.23). This effect may be caused by unreacted polymer chain end thiol groups forming disulfide bonds that increased the polymer molecular weight and distribution during the nanoparticle formation and lyophilization processes. After 4 min irradiation, the molecular weight remained above 2000 g mol^−1^ as the PEG block (∼2000 g mol^−1^) is non-photo degradable, indicating that the mixture obtained after irradiation mainly contains short oligomers of polyTNB and low molecular weight PEG-polyTNB. These shortened PEG-polyTNB fragments may still play a role as the stabilizer of nanoparticles, but the small fraction of PEG that completely cleaved from the hydrophobic polyTNB would release into solution, which explains the observed zeta potential decrease.

**Fig. 4:**
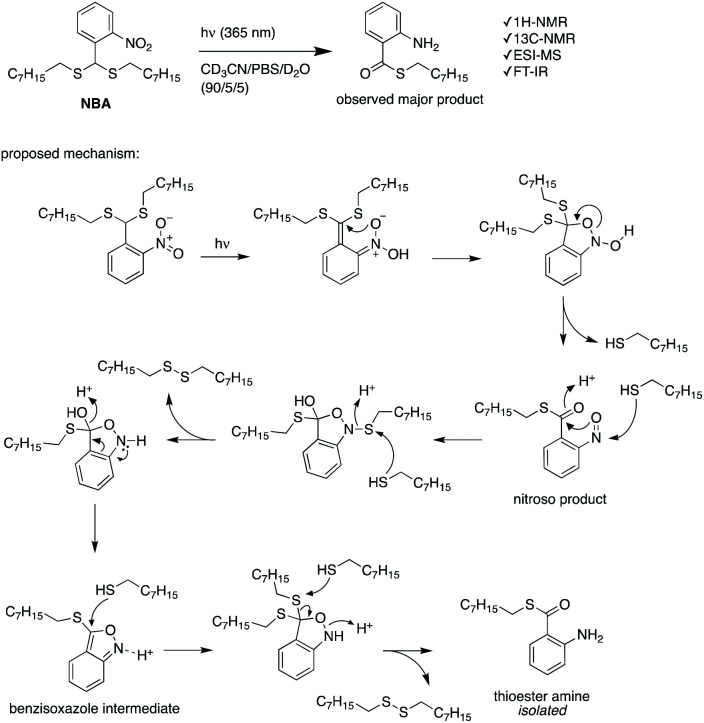
The photochemical cleavage of NBA, and a proposed reaction mechanism for the formation of the observed thioester amine product.

In an attempt to shed light on the mechanism of the photochemical thioacetal cleavage reaction, we synthesized a small molecule, ((2-nitrophenyl)methylene)bis(octylsulfane) (NBA, Fig. 4 and S5†), an analog of the TNB repeat unit. NBA contains the same thioacetal group as TNB, but has alkyl substituents on sulfur instead of the glycol units in TNB, to simplify the structure. Irradiation of NBA with UV-A (24 h) led to complete conversion of the NBA structure, as shown by the disappearance of the 5.49 ppm thioacetal proton peak in the ^1^H-NMR spectrum (Fig. S6†). After purification of the product mixture, we characterized the main product by ^1^H-NMR, FTIR and mass spectrometry (Fig. S7, S8 and S10†). Irradiation of *o*-nitrobenzyl derivatives will often lead to *o*-nitrosobenzyl products.^28^ In our case, however, the analytical data of the product did not align with the *o*-nitroso product. Instead, we found evidence suggesting the formation of an *o*-amino benzoic acid thioester (Fig. 4). FTIR (Fig. S10†) showed strong bands at 3475 and 3365 cm^−1^, suggesting the existence of a primary amine; mass spectrometry showed a main signal at *m*/*z* 266, 16 Da less than the benzisoxazole intermediate, indicating a dehydration process may have occurred. Having an amine product instead of the nitroso means a formal reduction. Building on a reported mechanism of photolysis of 1-(2-nitrophenyl)ethyl phosphates in the presence of thiol,^38^ we propose a mechanism as shown in Fig. 4. The mechanism proceeds to first give the common nitroso product, which is then further reduced to give the reported benzisoxazole intermediate and finally the amine product. In ^1^H-NMR and MS measurements we observed the formation of dioctyl disulfide, the typical oxidation product of octyl thiol (Fig. S6 and S9†). The large amount of dioctyl disulfide suggests that octyl thiol plays a role in the process of nitroso reduction, although this is by no means a clean process and other mechanisms may also be at play. In the polymer irradiation experiments, we do not observe the formation of high molecular weight disulfide polymers. This result suggests that the polymer breakdown either follows a partially different mechanism or that the formed thiols may be converted to low molecular weight cyclic or linear disulfide fragments.

### ROS-response of PEG-polyTNB nanoparticles

As thioketal and thioacetal groups have been reported as being labile to reactive oxygen species (ROS), we also assessed the sensitivity of the nitrobenzylthioacetal derived polymers to some ROS types. We incubated PEG-polyTNB nanoparticles in PBS solution with H_2_O_2_ (500 mM) or KO_2_ (100 mM) at 37 °C for 24 h, and analyzed the resulting mixtures after lyophilization by ^1^H-NMR. The results (Fig. S11†) show that the thioacetal structure was changed (new peaks appeared at *δ* = 7.67, 7.52 and 6.01 ppm) in 500 mM H_2_O_2_ but surprisingly remained stable in 100 mM KO_2_ and H_2_O_2_. These results demonstrated that PEG-polyTNB could respond to concentrated ROS, but proved much more stable than the often-reported dimethylene thioketal based polymers that disintegrate when exposed to H_2_O_2_ and KO_2_ at a concentration of 10 mM.^11,34,39^ This substantial difference might be due to the electron withdrawing property of the nitro group that elevated the oxidation potential of the thioacetal compound.

### Light-triggered cargo release from PEG-polyTNB nanoparticles

**Fig. 5:**
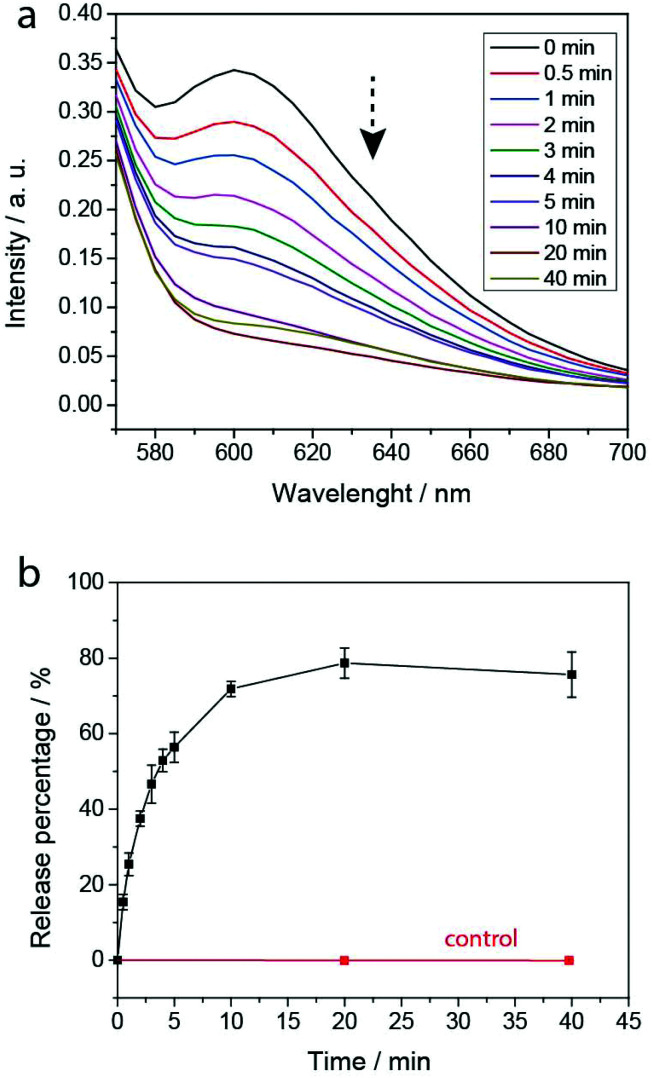
Nile Red release from NR-loaded nanoparticles in PBS under UV-A (365 nm) measured by fluorescence. (a) Nile Red fluorescence intensity after irradiation; (b) percentage of released Nile Red with increasing irradiation time; error bars represent the standard deviation of three measurements.

To evaluate the PEG-polyTNB nanoparticles as responsive carriers for drugs and other bioactive molecules, we first measured encapsulation and release of Nile Red as a hydrophobic drug model. Nile Red shows almost no fluorescence in water but can be intensely fluorescent in an apolar environment. The encapsulation efficiency (EE) of Nile Red in PEG-polyTNB nanoparticles was 42.6%, demonstrating a high hydrophobic cargo loading capacity of PEG-polyTNB nanoparticles. The dye release experiment was performed in PBS under UV-A (365 nm) at various time points (Fig. 5). The release rate decreased with irradiation time. When the nanoparticles were radiated for 0.5, 2, and 4 min, 15.4%, 37.5%, and 52.9% Nile Red were released, respectively. After 10 min continues irradiation, the Nile Red release process almost reached equilibrium at about 70–80%. And no release was observed without irradiation in the same period.

### Light-controlled cytotoxicity of PEG-polyTNB nanoparticles

**Fig. 6:**
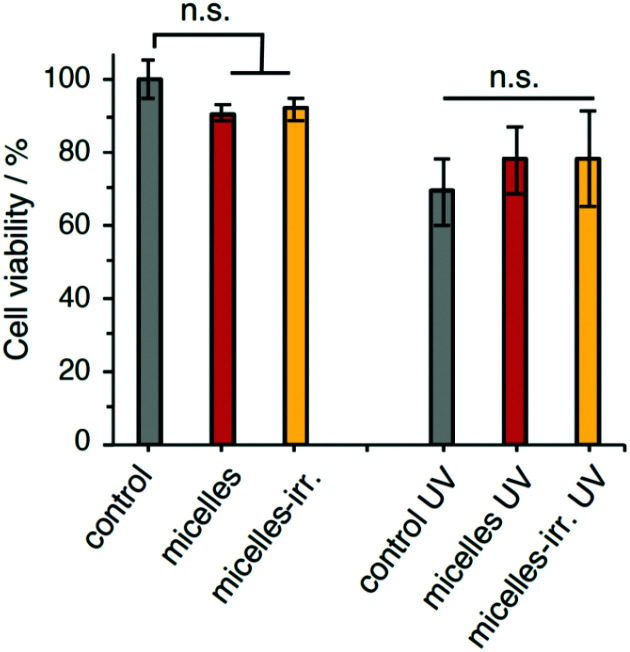
Viability of HeLa cells after 24 h with PEG-polyTNB nanoparticles and/or 30 s exposure to UV365 (90 mW cm^−2^). A control without treatment was considered 100% viability. n.s. = not significant; error bars are standard deviation (*N* = 4). In the micelles-irr. experiments, cells were incubated with polymer that had been previously irradiated with UV365 for 8 min.

The cytocompatibility of the empty nanoparticles was evaluated using a human cervix carcinoma cell line (HeLa). The survival fraction of HeLa cells was higher than 90% after incubation for 24 h at a micelle concentration of 0.25 mg mL^−1^ (Fig. 6). Likewise, incubation with irradiated nanoparticles showed over 90% cell viability. These results indicate that the polymeric nanoparticles and their fragments and irradiation products are biocompatible and do not show significant cytotoxicity. Irradiation of untreated cells (UV-A 365 nm, 90 mW cm^−2^, 30 s), showed a 30% reduction in viability. Cell viability after irradiation did not show significant changes when incubated with PEG-polyTNB nanoparticles, or with irradiated nanoparticles (micelles-irr.). Overall, these experiments show that these polymer nanoparticles do not affect cell viability, both in the absence and presence of UV-A irradiation.

## Conclusion

In conclusion, we report a facile method for the synthesis of photo-cleavable polymers having a photoresponsive moiety incorporated in the polymer backbone based on thioacetal formation with *o*-nitrobenzaldehyde. The thiol end group of polyTNB can be clicked to PEG, to form an amphiphilic block copolymer that can be employed for the assembly of nanoparticles. The nanoparticles show fast release of drug model Nile Red (>50%) in 5 min when exposed to UV-A light. Moreover, the polyTNB nanoparticles, unlike other reported polymers containing thioacetal bonds, are resistant to high concentrations of ROS. For example, the structure kept stable over 24 h in environments containing up to 100 mM KO_2_ or H_2_O_2_. Lastly, we demonstrated the PEG-polyTNB nanoparticles are biocompatible. We expect that this easily synthesized photocleavable polymer can be used as a drug carrier for photodynamic therapy by combination with other energy up-conversion nanoparticles.^40^

## Author contributions

Y.M. synthesized the polymers and characterized the nanoparticles, T.G.B. performed the small molecule experiments, H.L. performed cell experiments. All authors contributed to analysing data and commented on the manuscript. Y.M. wrote the manuscript. R.E. and A.G.D. conceived and supervised the project.

## Conflicts of interest

There are no conflicts to declare.

## Supplementary Material

PY-012-D1PY00514F-s001
