# Supplementary material for: Photo cleavable thioacetal block copolymers for controlled release
Source: Polym Chem. 2021 Jun 8;12(25):3612–8. doi: 10.1039/d1py00514f (PMC8240465; doi:10.1039/d1py00514f)
Supplement: PY-012-D1PY00514F-s001 [file PY-012-D1PY00514F-s001.pdf]

Electronic Supplementary Material (ESI) for Polymer Chemistry.
This journal is © The Royal Society of Chemistry 2021

Supporting Information

Photo cleavable thioacetal block copolymers for controlled release

Yongjun Men[a]‡, Tobias G. Brevé[a]‡, Huanhuan Liu[a,b]‡, Antonia G. Denkova[b] and Rienk Eelkema[a]**

[a]Department of Chemical Engineering, Delft University of Technology, van der Maasweg 9, 2629 HZ Delft, The Netherlands.

[b]Department of Radiation Science and Technology, Delft University of Technology, Mekelweg 15, 2629 JB Delft, The Netherlands.

Characterization

The chemical structures of the synthetic molecules and polymers were characterized by ^{1}H NMR spectroscopy (Agilent-400 MR DD2 NMR spectrometer, 399.67 MHz for ^{1}H and 100.5 MHz for ^{13}C), using D_2O, acetonitrile-d3, DMSO-d6, Chloroform-d as solvent. The molecular weights of the polymers were measured with a Shimadzu Prominence GPC system equipped with a PL gel 5 μm mixed D column (Polymer Laboratories) and differential refractive index and UV (254 nm) detectors. THF was used as eluent with a flow rate of 1 mL/min. A dynamic light scattering (DLS) spectrometer (Malvern Zetasizer Nano ZS) was employed to characterize the diameter and distribution of the polymer micelles. A TEM JEOL 2100 electron microscope at an acceleration voltage of 60 kV was used for electron microscopy imaging. TEM samples were prepared in the following way: a solution of a sample (6 μL) was air-dried on a carbon-coated Cu TEM grid (200 mesh). Drug loading content and drug-releasing amount were determined by fluorescence spectroscopy. The thermal analysis of polyTNB was carried out using Mettler Differential Scanning Calorimetry (DSC). A temperature ranging from -80 °C to 80 °C with a heating rate of 10 °C/min, a nitrogen gas flow rate of 50 mL/min, and a weighted sample of 6.210 mg are the conditions used in this analysis. GS-MS samples were analyzed using an Agilent 5977 GC/MSD equipped with a Stabilwax MS column (oven temperature: 250 °C, flow: 2.5 ml/min). LCMS measurements were performed using LTQ XL spectrometer equipped with Shimadzu HPLC setup operating at 0.2 mL/min flow rate with water/MeCN mobile phase containing 0.1%vol formic acid and Discovery C18 column.

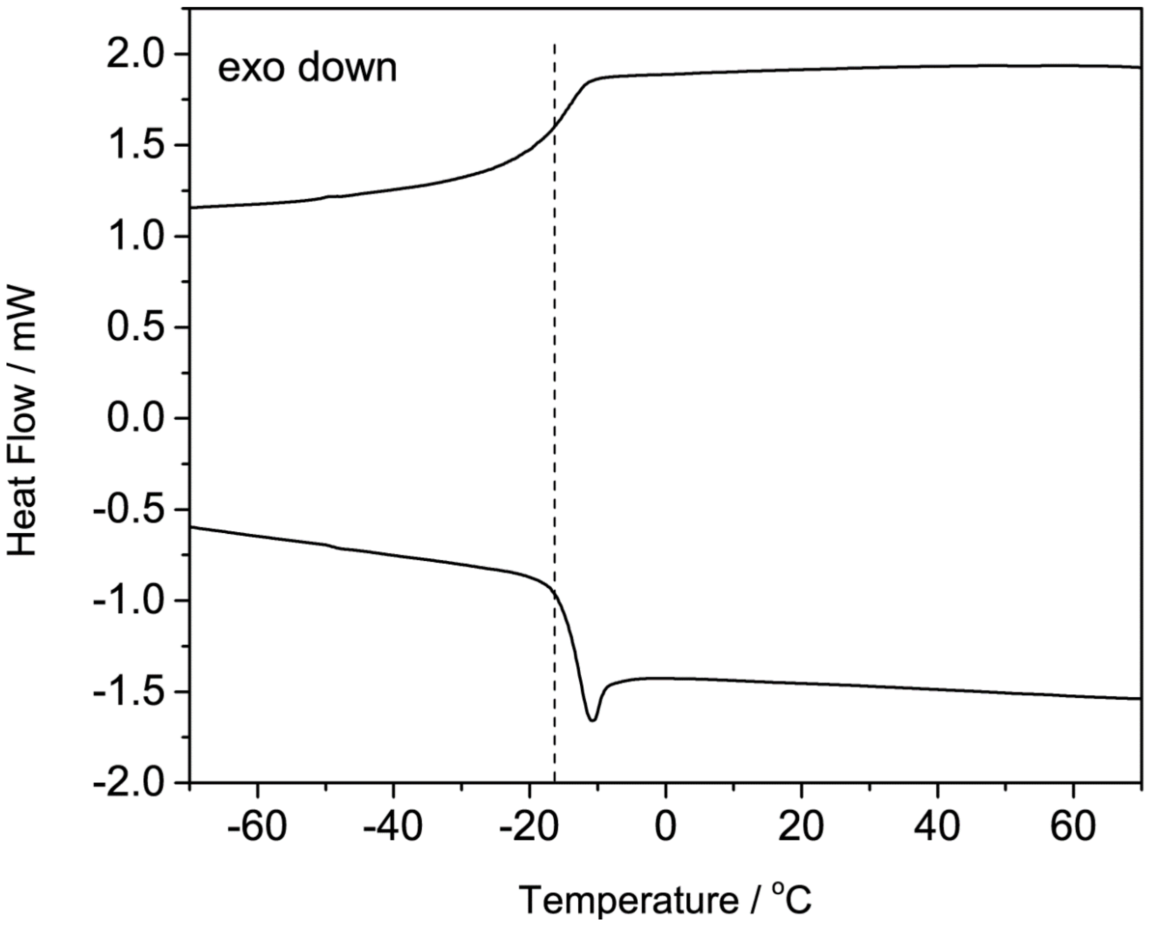

Figure S1. T_g of polyTNB measured by DSC.

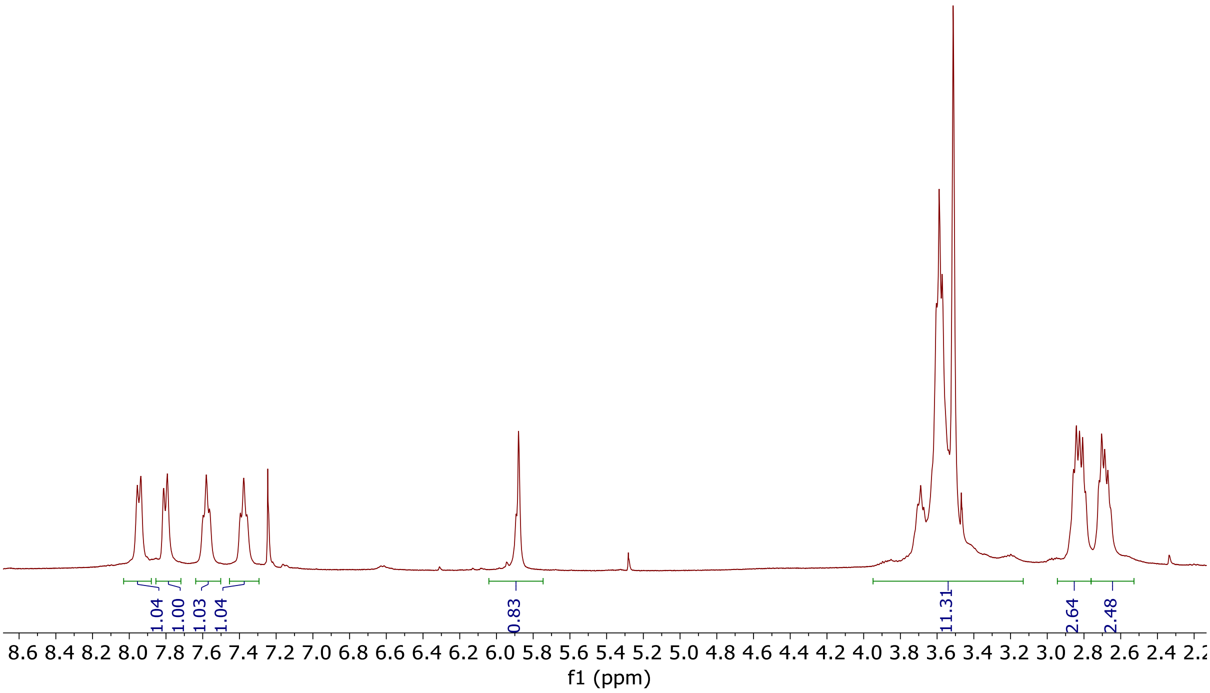

Figure S2. ^{1}H-NMR ($CDCl_3$, 400MHz) spectrum of polyTNB.

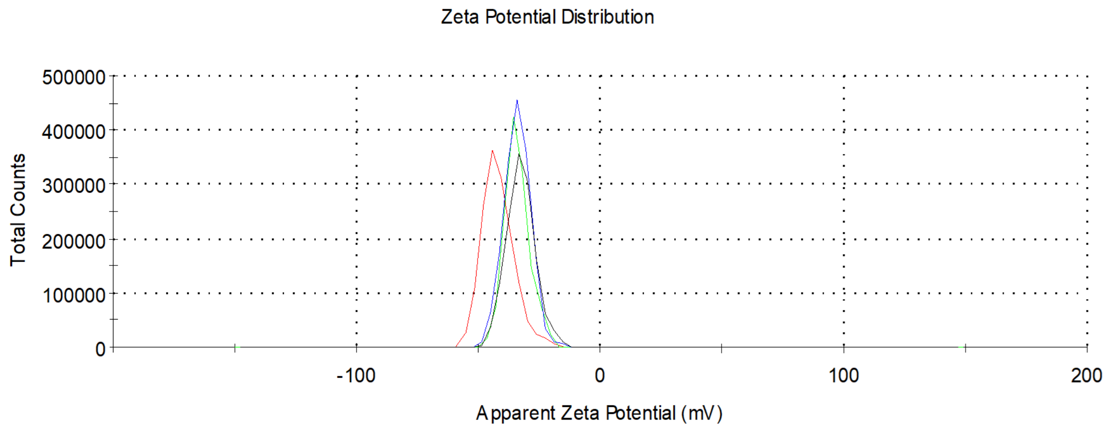

Figure S3. Zeta potential distribution of PEG-polyTNB nanoparticles after UV = 0 min (red line), 0.5 min (green line), 2 min (blue line), 4 min (black line) radiation.

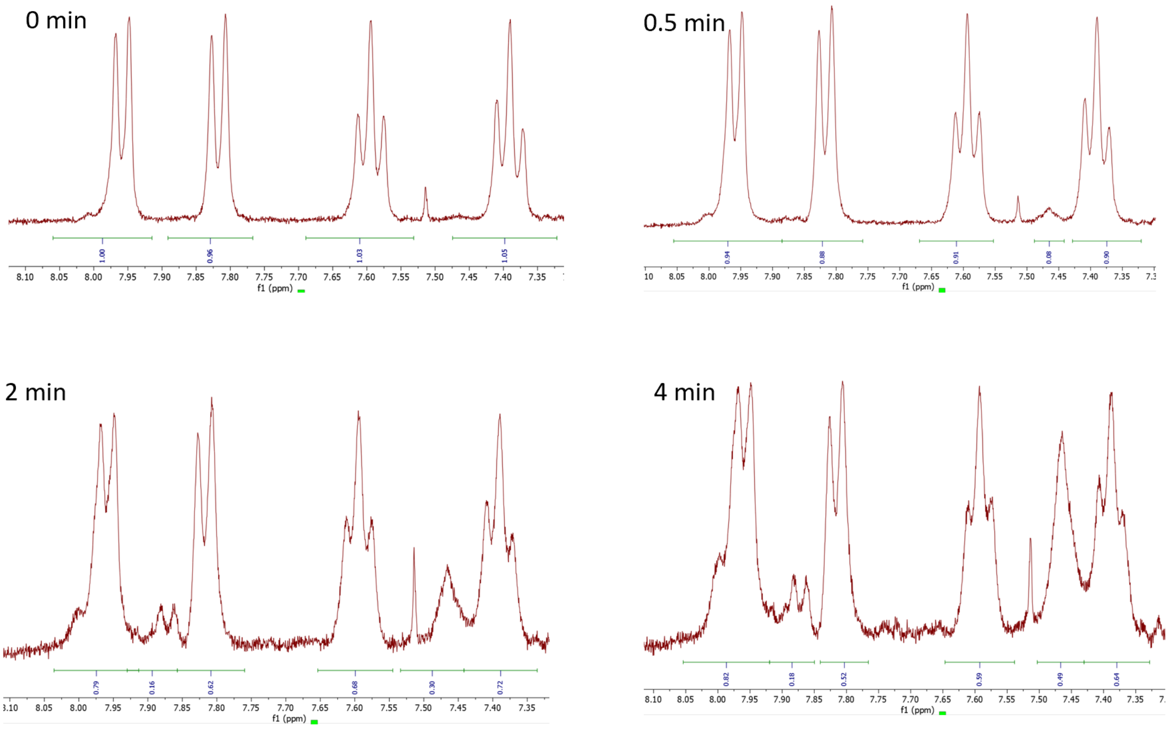

Figure S4. ^{1}H-NMR (400MHz, $CDCl_3$) spectra of PEG-polyTNB nanoparticles after UV = 0, 0.5, 2, 4 min irradiation.

Synthesis of ((2-nitrophenyl)methylene)bis(octylsulfane) (NBA)

1-Octanethiol (0.843 g, 10 mmol), *o*-nitrobenzaldehyde (0.755 g, 5 mmol), and *p*-toluenesulfonic acid (0.9 mg, 5 µmol) was loaded into a 10 mL single neck round-bottom flask containing a stirring bar. The reaction was performed at 80 °C for 48 h under argon atmosphere. The crude product was purified over silica (100% PE to 95:5 PE:EtOAc), the product fractions were collected and concentrated. This gave the title compound as a colourless oil. Yield: 82 % (1.31 g, 4.1 mmol). ^{1}H NMR (400 MHz, $CDCl_3$) δ = 7.98 (d, **3**, J = 7.99, 1H), 7.80 (d, **6**, J = 7.99, 1H), 7.60 (t, **4**, J = 7.99, 1H), 7.38 (t, **5**, J = 7.99, 1H), 5.70 (s, **7** , 1H), 2.60 (m, **10/18**, 4H), 1.54 (m, **11/19**, 4H), 1.24 (m, **12-16/20-24**, 20H), 0.86 (t, **17/25**, J = 7.99 Hz, 6H). ^{13}C NMR (100 MHz, $CDCl_3$) δ = 148.02 (**2**), 136.13 (**1**), 133.12 (**4**), 130.57 (**3**), 128.28 (**5**), 124.22 (**6**), 46.86 (**7**), 32.85 (**10**/**18**), 31.76 (CH_2, aliphatic), 29.11 (CH_2, aliphatic), 29.07 (CH_2, aliphatic), 28.78 (CH_2, aliphatic), 22.61 (CH_2, aliphatic), 14.06 (**17**/**25**). m/z calc.: 425.24, found: 426.15 $[M]^+$

^{1}H-NMR

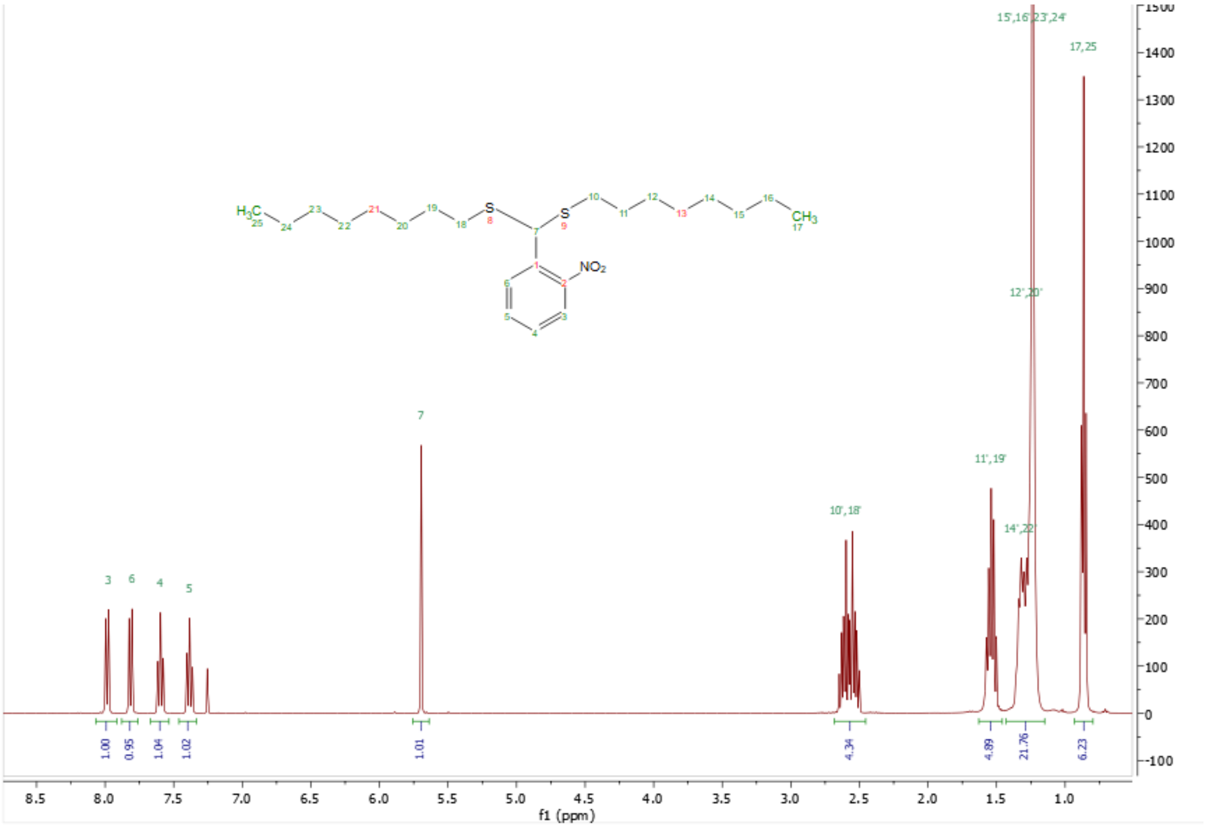

H-H COSY

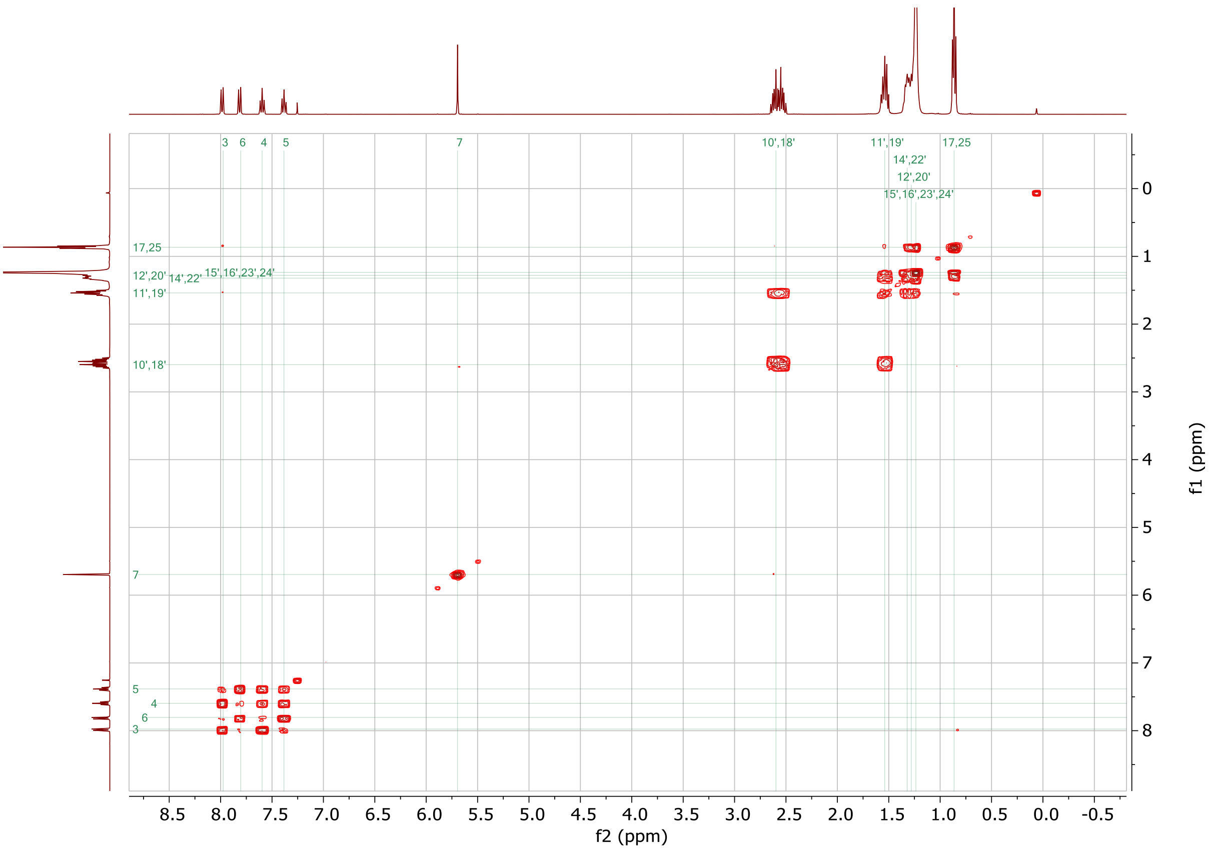

^{13}C-NMR

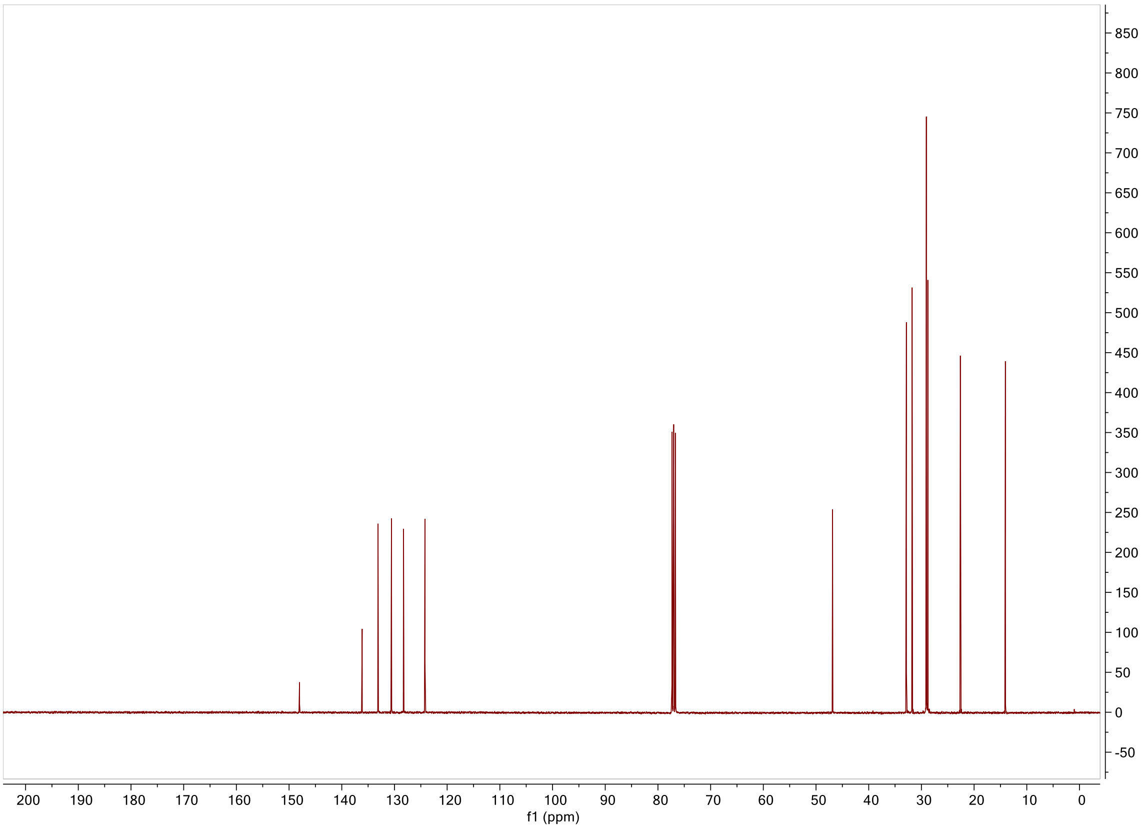

HSQC

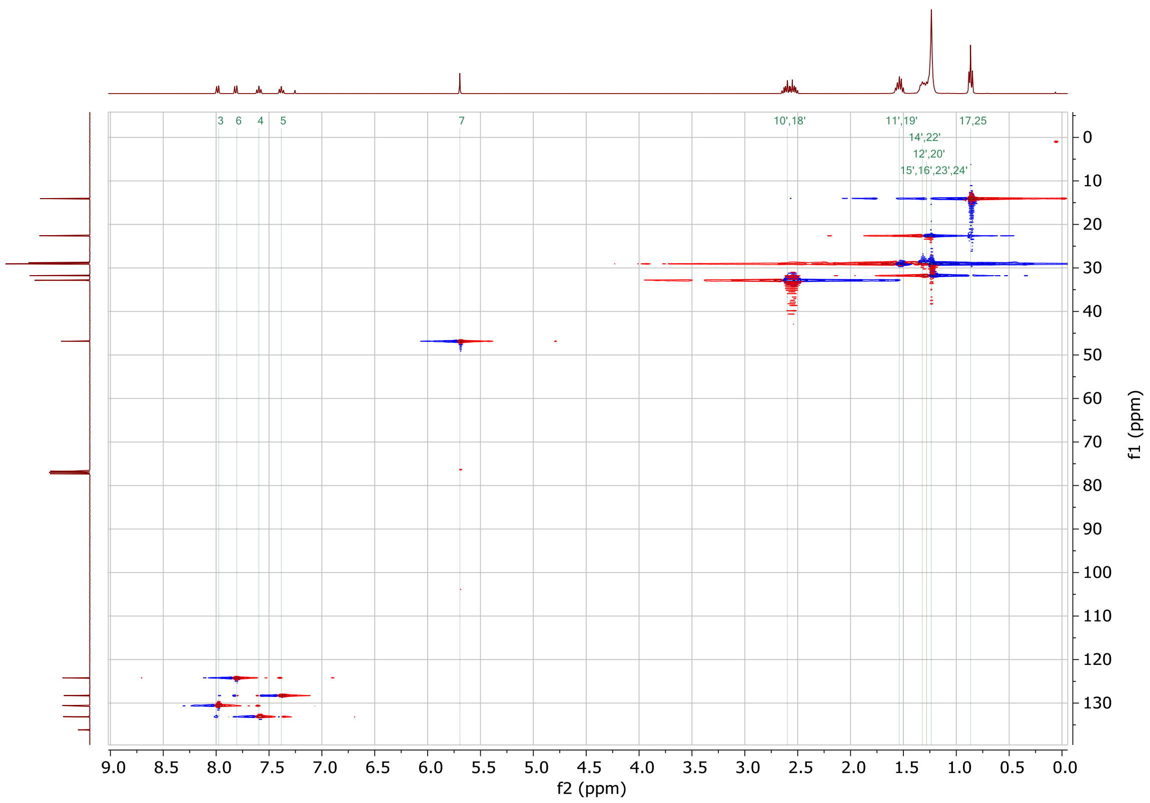

Figure S5 ^{1}H-NMR, H-H COSY, ^{13}C-NMR and HSQC of NBA in $CDCl_3$.

Light induced degradation of model compound NBA

Model compound NBA was dissolved in acetonitrile-d3 and irradiated with 365nm light in order to examine the photodegradation over time. The top spectrum (t = 0 min., Figure **S6**) shows NBA. Upon irradiation with 365nm light the signals belonging to NBA start to disappear, indicating the degradation of NBA. The formation of the thioester can be observed (corresponding signals are indicated with black stars in the final spectrum). The formation of the amine thioester product is confirmed by ESI-MS analysis (found: m/z 266.12; mass calc.: 266.16 $[M]^{+1}$). Also, the formation of dioctyl disulfide is observed as the CH_2 signals directly next to the sulfur atoms appear at 2.47 ppm (red stars). GC-MS analysis, showing m/z 290.2 (mass calc.: 290.21 [M]), confirms the formation of dioctyl disulfide (Figure **S9**). The formation of the thioester photoproduct is further supported by an additional photocleavage experiment, in which the reaction mixture after 365nm light irradiation was concentrated and re-dissolved in $CDCl_3$. The ^{1}H-NMR spectrum in figure **S8** shows that the resonances belonging to the thioester photoproduct match with the literature values of thioester analog S-butyl 2-aminobenzothioate.[1]

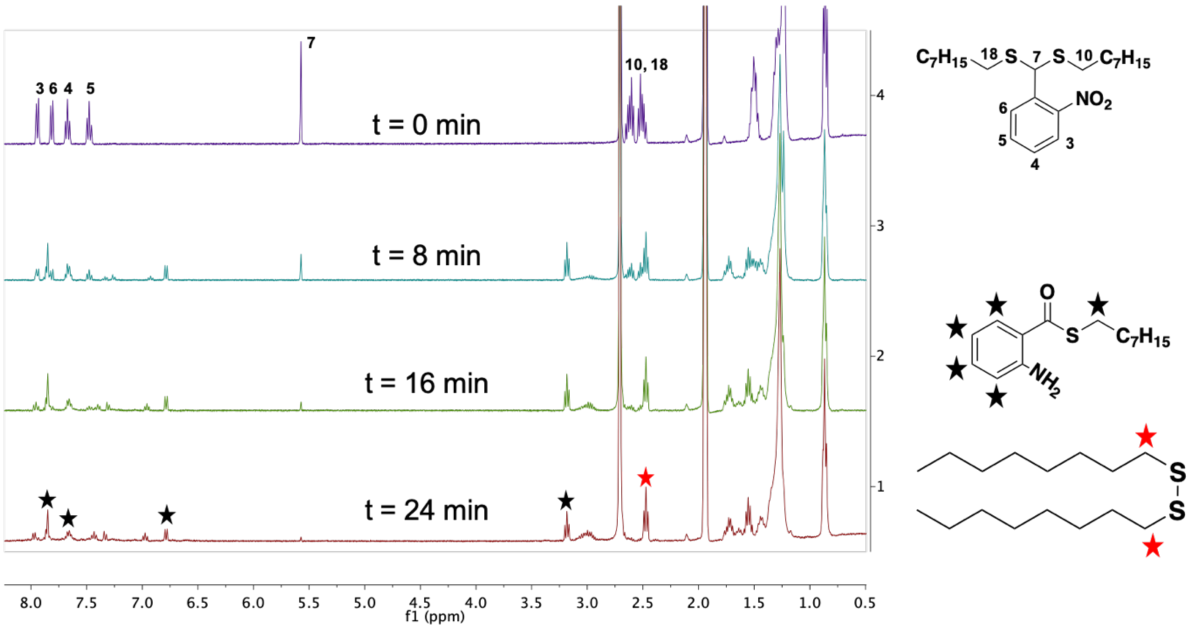

Figure S6 365 nm irradiation experiment of **NBA** monitored by ^{1}H-NMR ($CDCl_3$). Time points on which samples were taken and analyzed are indicated. Black stars indicate resonances belonging to the thioester photoproduct shown in Figure **S8**. Red stars indicate resonances belonging to dioctyl-disulfide (mass shown in Figure **S9**).

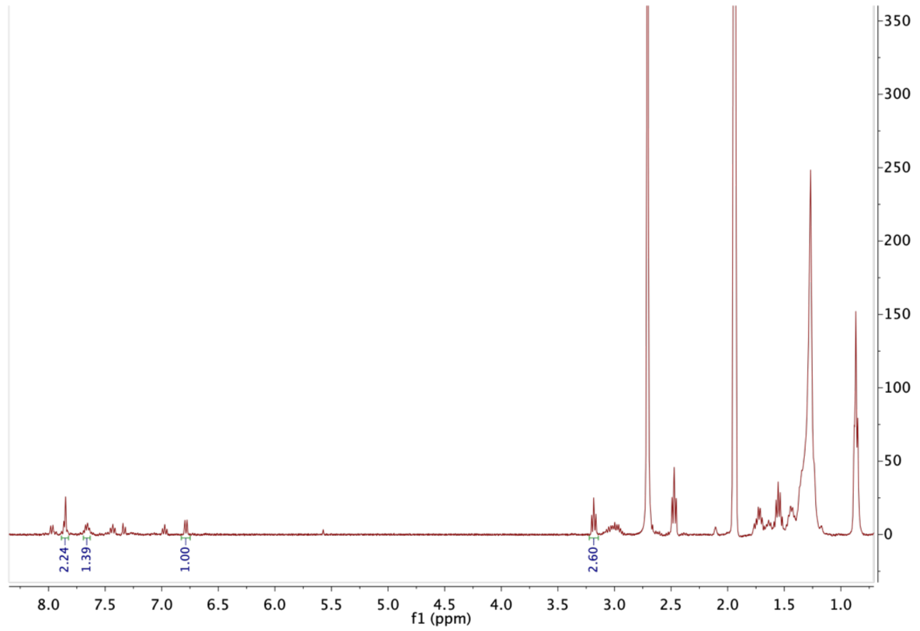

Figure S7 the integrated 1H-NMR spectrum taken after 24 minutes of 365 nm light irradiation (Figure **S6**), showing the aromatic signals and S-C*H*2 of the thioester photoproduct.

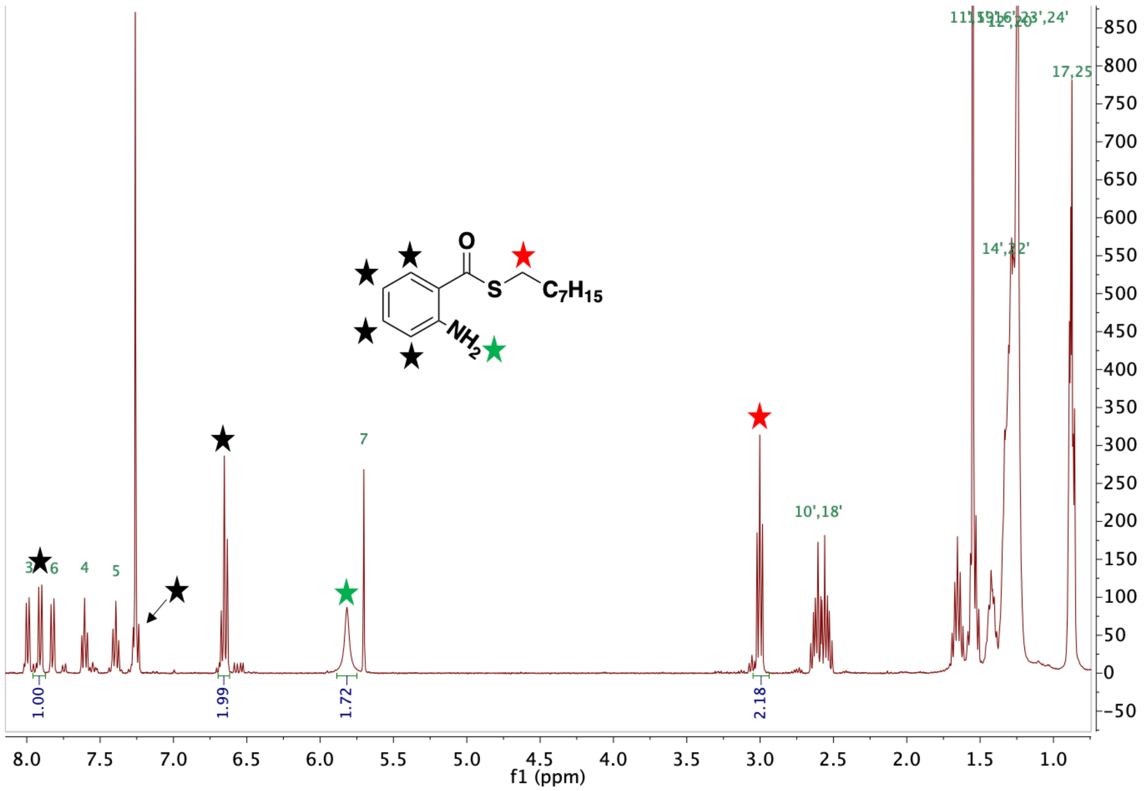

Figure S8 ^{1}H-NMR ($CDCl_3$) spectra of the thioester photoproduct. Resonances indicated with colored stars match with reported values of thioester analog S-butyl 2-aminobenzothioate.[1] Resonances numerically labeled belong to NBA (Figure **S5**).

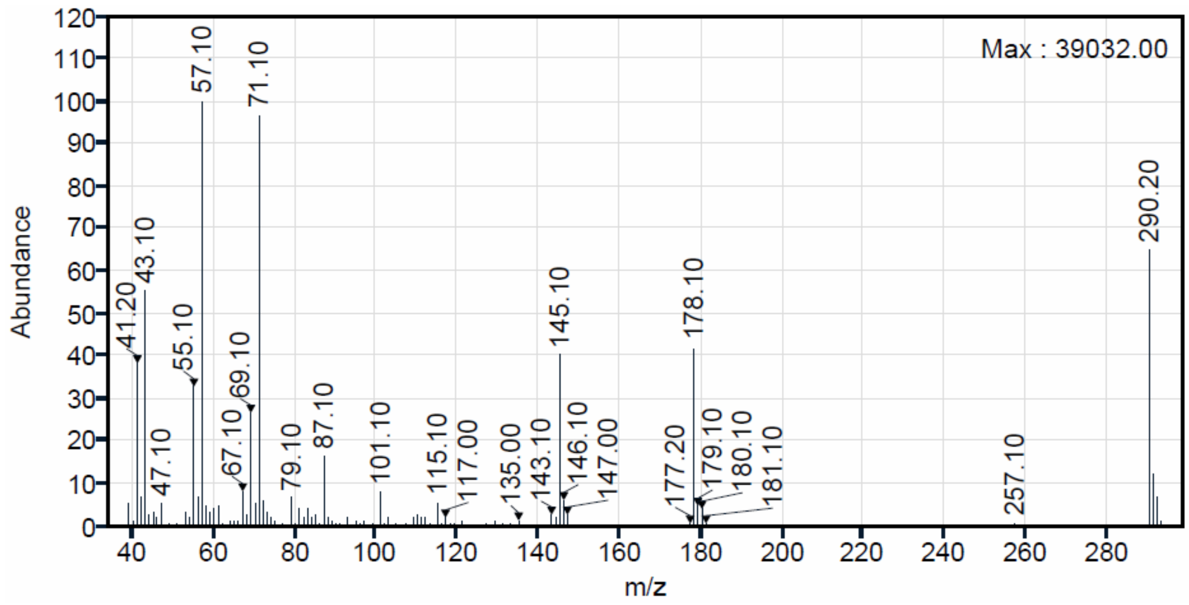

Figure S9 GC-MS spectrum obtained after photocleavage of NBA. Mass of dioctyl-disulfide (mass calc.: 290.20) is detected.

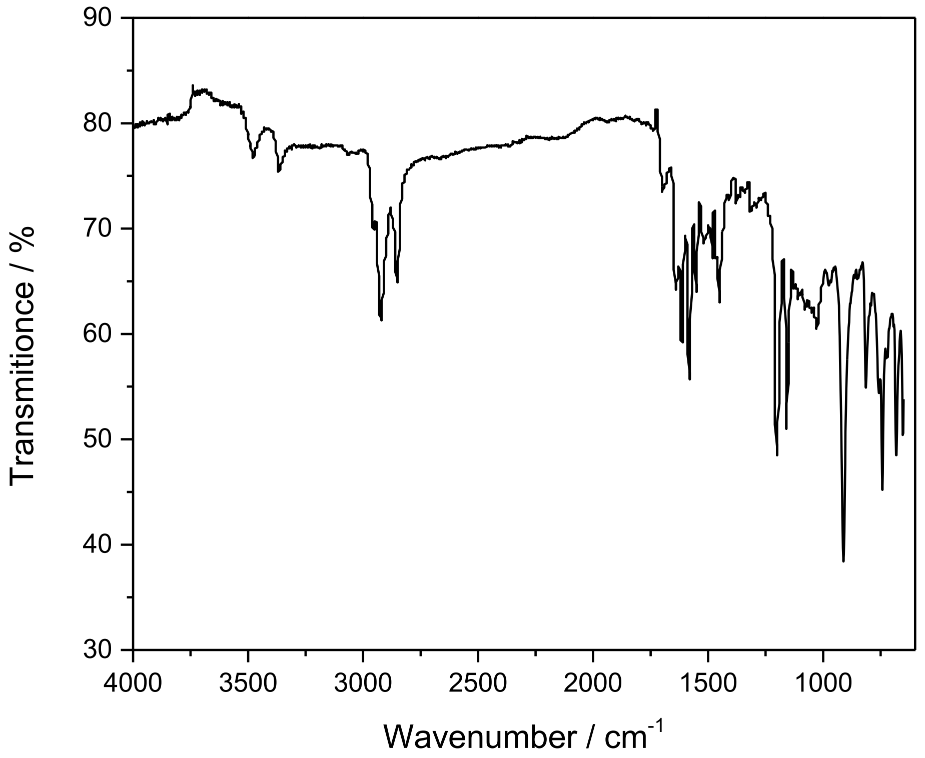

Figure S10 FTIR spectrum of the decomposition products of NBA.

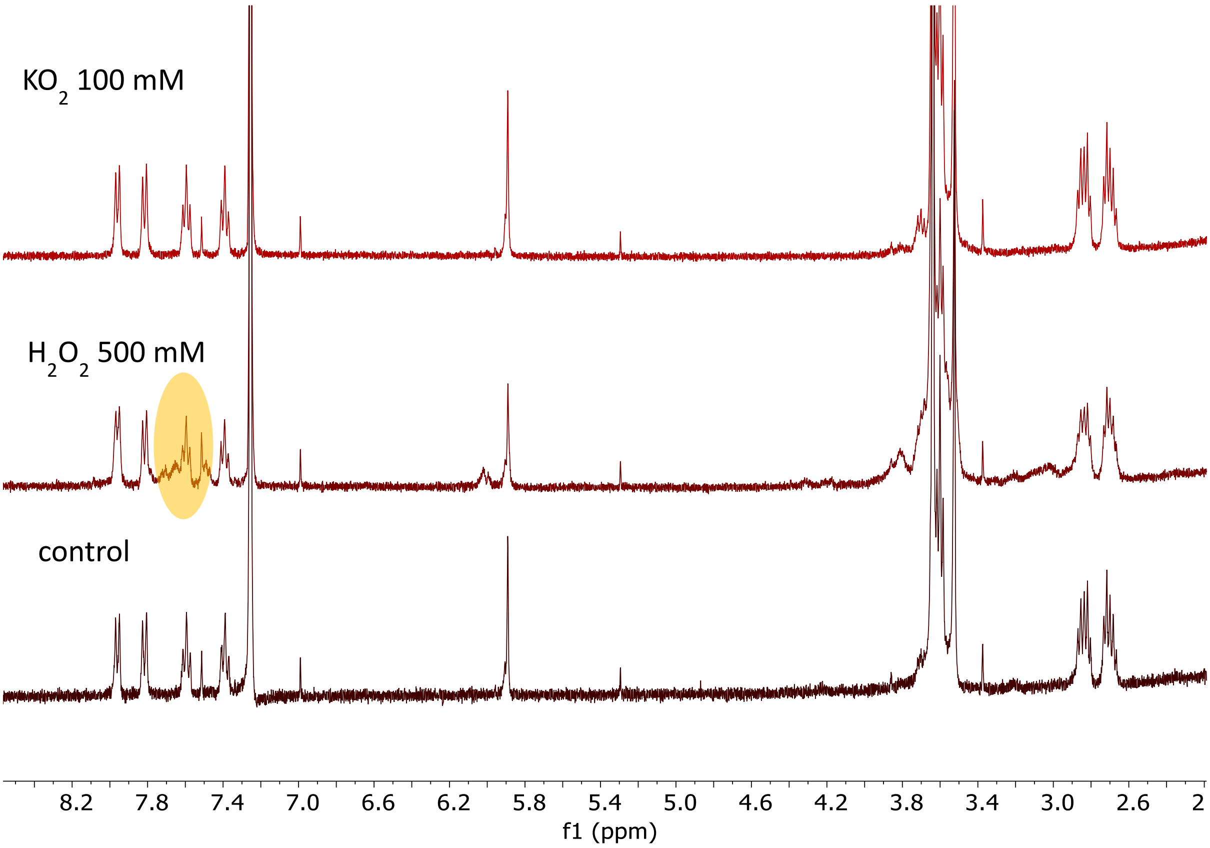

Figure S11. ^{1}H-NMR of PEG-polyTNB nanoparticles were incubated in PBS solution with H_2O_2 (500 mM) or KO_2 (100 mM) at 37 °C for 12 h.

1. Poudel, T. N.; Khanal, H. D.; Lee, Y. R., Base-promoted ring opening of 3-chlorooxindoles for the construction of 2-aminoarylthioates and their transformation to quinazolin-4(3H)-ones. *New J. Chem.* **2018,** *42*, 4735-4741.
